# ShuffleNetV2 SSM MLCA: a lightweight recognition network for wheat fungal diseases

**DOI:** 10.3389/fpls.2026.1841159

**Published:** 2026-07-08

**Authors:** Yuxin Shi, Cheng Zeng, Nan Chi

**Affiliations:** 1 School of Mathematics and Statistics, Guizhou University, Guiyang, China; 2 College of Science, Guizhou Institute of Technology, Guiyang, China; 3 College of Innovation and Entrepreneurship,Guizhou Institute of Technology, Guiyang, China; 4 The Key Laboratory of New Power System Operation Control of Guizhou Province, Guiyang, China; 5 Special Key Laboratory of Artificial Intelligence and Intelligent Control of Guizhou Province, Guiyang, China

**Keywords:** lightweight model, wheat fungal diseases, ShuffleNetV2, SS-Conv-SSM, half convolution, mixed local channel attention

## Abstract

**Introduction:**

Wheat is one of the most widely planted staple crops worldwide and underpins global food security. Fungal diseases severely threaten wheat growth and trigger massive yield losses during cultivation. Traditional manual diagnosis is time-consuming and highly subjective, while existing deep learning models often struggle to achieve high accuracy and robustness in complex field environments. Accurate identification of these fungal diseases is therefore vital to secure grain production.

**Methods:**

This paper constructs a lightweight convolutional neural network named ShuffleNetV2_SSM_MLCA for wheat fungal disease classification. First, the original basic blocks of ShuffleNetV2 are substituted with SS-Conv-SSM modules to strengthen the extraction of fine-grained lesion features amid visually analogous fungal disease samples; half convolution is embedded to cut down model computational overhead. Second, a Mixed Local Channel Attention (MLCA) unit is attached to the convolution branch of each SS-Conv-SSM module, which adaptively highlights discriminative disease features and filters irrelevant background noise. Standard training configurations and five-fold cross-validation are adopted for fair model evaluation.

**Results:**

Comparative experiments reveal that the presented network reaches a classification accuracy of 91.35%, which surpasses the original ShuffleNetV2 baseline by 1.16 percentage points. Controlled ablation tests verify the independent performance gain of each core component: the SS-Conv-SSM module raises overall accuracy by 0.89%, and the MLCA mechanism brings an extra 0.27% accuracy increment.

**Discussion:**

The proposed ShuffleNetV2_SSM_MLCA architecture strikes a favorable trade-off between model lightweight property and classification performance. It delivers a low-computation, high-precision recognition scheme for wheat fungal diseases and lays a solid technical foundation for real-time disease monitoring in intelligent agricultural scenarios.

## 1 Introduction

Wheat is one of the most widely cultivated staple food crops worldwide and plays a vital role in ensuring food security and supporting agricultural economies across many countries and regions. Rich in protein, minerals, vitamins, dietary fiber, and other nutrients, wheat serves as a major source of energy and nutrition for humans Khalid et al. (2023). However, wheat is susceptible to a wide range of diseases throughout its growth cycle. Among them, fungal diseases, including rust, leaf spot, and Fusarium head blight, are particularly destructive Vagelas (2025). Due to their high infectivity and adaptability to diverse environmental conditions, these diseases can cause substantial yield losses and quality deterioration when outbreaks occur on a large scale. Previous studies have shown that fungal diseases can reduce cereal production by 15%–20%, while yield losses may exceed 50% in severely affected regions Różewicz et al. (2021). Recent research further indicates that five major fungal pathogens alone are responsible for annual global wheat yield losses exceeding 62 million tons. In extreme cases, such losses may directly threaten the food supply of hundreds of millions of people Chai et al. (2022).

Climate change, together with agricultural practices such as maize–wheat rotation and conservation tillage, has accelerated pathogen evolution and facilitated transboundary disease dissemination. Highly virulent pathotypes, exemplified by Ug99 stem rust, have spread across continents through atmospheric circulation, while Fusarium head blight has expanded rapidly throughout sub-Saharan Africa, particularly in Ethiopia. These disease outbreaks result in substantial yield losses and significant accumulation of deoxynivalenol (DON) in harvested grain, thereby posing major threats to global food and feed security Singh et al. (2025); Horo et al. (2025). Beneficial microorganisms, particularly species of Bacillus and Trichoderma, offer considerable promise as environmentally sustainable alternatives for disease control; however, inconsistent efficacy under field conditions remains a major obstacle to their widespread adoption Kashyap et al. (2025). Given the escalating challenges posed by yield losses, mycotoxin contamination, and the emergence of fungicide resistance, accelerating research efforts and promoting the field-scale implementation of effective disease management strategies are imperative, highlighting the critical role of integrated pest management in sustainable wheat production.

Traditional field-based disease identification primarily relies on manual inspection, which is not only inefficient but also susceptible to subjective judgment, leading to poor consistency and reliability in recognition results. To address the limitations of manual diagnosis, machine learning techniques have gradually been introduced into crop disease identification research worldwide Bhola and Kumar (2025); Mehra et al. (2016). Most conventional machine learning approaches rely on manually engineered features for disease recognition. Commonly extracted features include color characteristics Bao et al. (2021), hyperspectral information Yuan et al. (2019), and texture features Shafi et al. (2021); Ahmed and Yadav (2023), while some studies have explored disease identification by integrating multiple feature types Khan et al. (2022). For example, Zhuang et al. (2025) employed a continuous wavelet projection algorithm to select hyperspectral features and combined it with a k-nearest neighbor classifier. Two characteristic wavelengths, 668 nm and 894 nm, were identified, enabling the classification of wheat powdery mildew, stripe rust, and leaf rust with an overall accuracy of 77%. This approach demonstrated advantages in feature reduction and computational efficiency. However, such methods heavily depend on expert knowledge for manual feature engineering. Consequently, their generalization capability is often limited, making it difficult to maintain robust performance across different environments and disease conditions.

With the rapid advancement of deep learning technologies, convolutional neural networks (CNNs) have been widely employed in image classification tasks. Compared with traditional machine learning approaches, CNNs eliminate the need for manual feature engineering and are capable of automatically learning hierarchical feature representations from raw data, thereby significantly improving the ability to process complex image information. Consequently, CNN-based methods have been extensively applied to plant disease identification. Uğuz and Uysal (2021) combined a customized CNN with VGG-based transfer learning for olive leaf disease classification. By incorporating data augmentation and algorithmic optimization strategies, the proposed method achieved an accuracy of 95% on a dataset containing 3,400 samples, and a corresponding web application was subsequently developed. Pandian et al. (2022) developed a five-layer CNN and achieved a classification accuracy of 98.41% for 26 categories of plant diseases on a dataset comprising 234,000 images after data augmentation and hyperparameter optimization. Geetharamani and Pandian (2019) designed a nine-layer CNN and reported an accuracy of 96.46% on a dataset covering 39 classes of leaf images. Sachdeva et al. (2021) integrated Bayesian learning with deep CNNs and achieved an accuracy of 98.9% in recognizing 15 plant diseases using the PlantVillage dataset. Ahila Priyadharshini et al. (2019) improved the LeNet architecture and employed PCA whitening together with data augmentation techniques to classify four maize leaf diseases, achieving a final recognition accuracy of 97.89%.

Although deep convolutional neural networks have demonstrated outstanding performance in disease recognition tasks, conventional architectures are often characterized by substantial computational complexity and large numbers of parameters. To alleviate these limitations, a variety of lightweight network architectures, including the MobileNet series Howard et al. (2017); Sandler et al. (2018); Howard et al. (2019), ShuffleNet series Zhang et al. (2018); Ma et al. (2018), MnasNet Tan et al. (2019), GhostNet Han et al. (2020), and MobileOne Vasu et al. (2023), have been proposed to reduce model size and computational overhead. In recent years, these lightweight architectures have been extensively applied to crop disease recognition, including wheat disease detection. Nigam et al. (2024) developed a wheat rust recognition model by incorporating the CBAM attention mechanism into EfficientNet-B0. The proposed model achieved an accuracy of 98.70% and an F1-score of 98% on the WheatRust21 dataset, demonstrating enhanced capability in extracting disease-related features. Tyagi et al. (2024) integrated the CLAHE image enhancement algorithm with HSV-K-means segmentation to construct a lightweight convolutional network and further developed an Android-based application. The proposed approach achieved an accuracy of 99% in rice leaf disease detection, highlighting its practical applicability. Fang et al. (2023) presented a lightweight multi-scale network, termed IRCE, by integrating residual connections, Inception modules, and multiple attention mechanisms. With only 4.24 million parameters, the model achieved an accuracy of 98.7% and outperformed both conventional CNNs and mainstream lightweight architectures. Zhang et al. (2023) introduced an improved multi-scale module based on the VGG architecture for rice disease recognition. While maintaining a compact model size, the proposed method required only 26.1 MB of storage and exhibited substantially improved inference speed. Islam et al. (2024) combined EfficientNet-B3 with a spatial attention mechanism to develop a lightweight disease recognition model. Experimental results showed accuracies exceeding 96% on two wheat disease datasets, indicating a favorable balance between computational efficiency and recognition performance. Wen et al. (2023) conducted a comparative study of several lightweight architectures, including MnasNet. Through optimized training strategies, the best-performing model achieved an accuracy of 98.65% in wheat leaf disease recognition under complex field conditions while maintaining a parameter count of 19.09 million. Jouini et al. (2024) proposed a hybrid CropNet model for wheat disease recognition. The model yielded an accuracy of 99.80% on a dataset containing 5,000 samples; however, its size reached 51 MB with approximately 6 million parameters. Mookkandi et al. (2025) designed a lightweight MaxViT-based model by combining diverse convolutional structures with attention mechanisms. Despite containing only 814.7 thousand parameters, the model achieved an accuracy of 92.8% on a wheat disease dataset, demonstrating strong robustness to interference and suitability for deployment on edge devices.

The aforementioned studies demonstrate the effectiveness of deep learning in wheat disease classification. However, on one hand, the majority of existing research relies on datasets with limited disease categories, which poses a significant challenge for real-world application of the models. On the other hand, On the other hand, some models feature redundant architectures with large parameter sizes and high computational costs. Therefore, considering both aspects, future research should be directed toward enhancing model generalization capability with high-quality data, exploring more effective data augmentation techniques, and designing suitable lightweight modules—all aimed at achieving intelligent wheat disease diagnosis with high accuracy and low latency.

The main contributions of this paper are summarized as follows:

1. A wheat fungal disease dataset was constructed based on a publicly available wheat disease dataset collected from Kaggle. From the original dataset containing multiple disease categories, eight fungal diseases and healthy wheat samples were selected, resulting in a total of 9,210 images. This dataset provides a representative benchmark for evaluating wheat fungal disease classification under field conditions.
2. An improved lightweight wheat disease recognition framework, termed ShuffleNetV2_SSM_MLCA, was developed based on ShuffleNetV2. To enhance feature representation, the original building blocks of ShuffleNetV2 were replaced with SS-Conv-SSM modules, which effectively combine convolutional operations and state-space modeling to capture both local spatial information and long-range dependencies.
3. To alleviate the increase in computational complexity introduced by the SS-Conv-SSM modules, a half convolution strategy was incorporated into the network. This adaptation reduces computational cost and parameter redundancy while maintaining effective feature extraction capability, thereby achieving a better balance between recognition performance and model efficiency.
4. A Mixed Local Channel Attention (MLCA) mechanism was integrated into the convolution branch of the SS-Conv-SSM module to enhance the representation of disease-related discriminative features and suppress redundant background information. The combination of SS-Conv-SSM and MLCA further improves the model’s ability to distinguish visually similar wheat fungal diseases.

## 2 Materials and method

### 2.1 Wheat disease dataset

**Figure 1:**
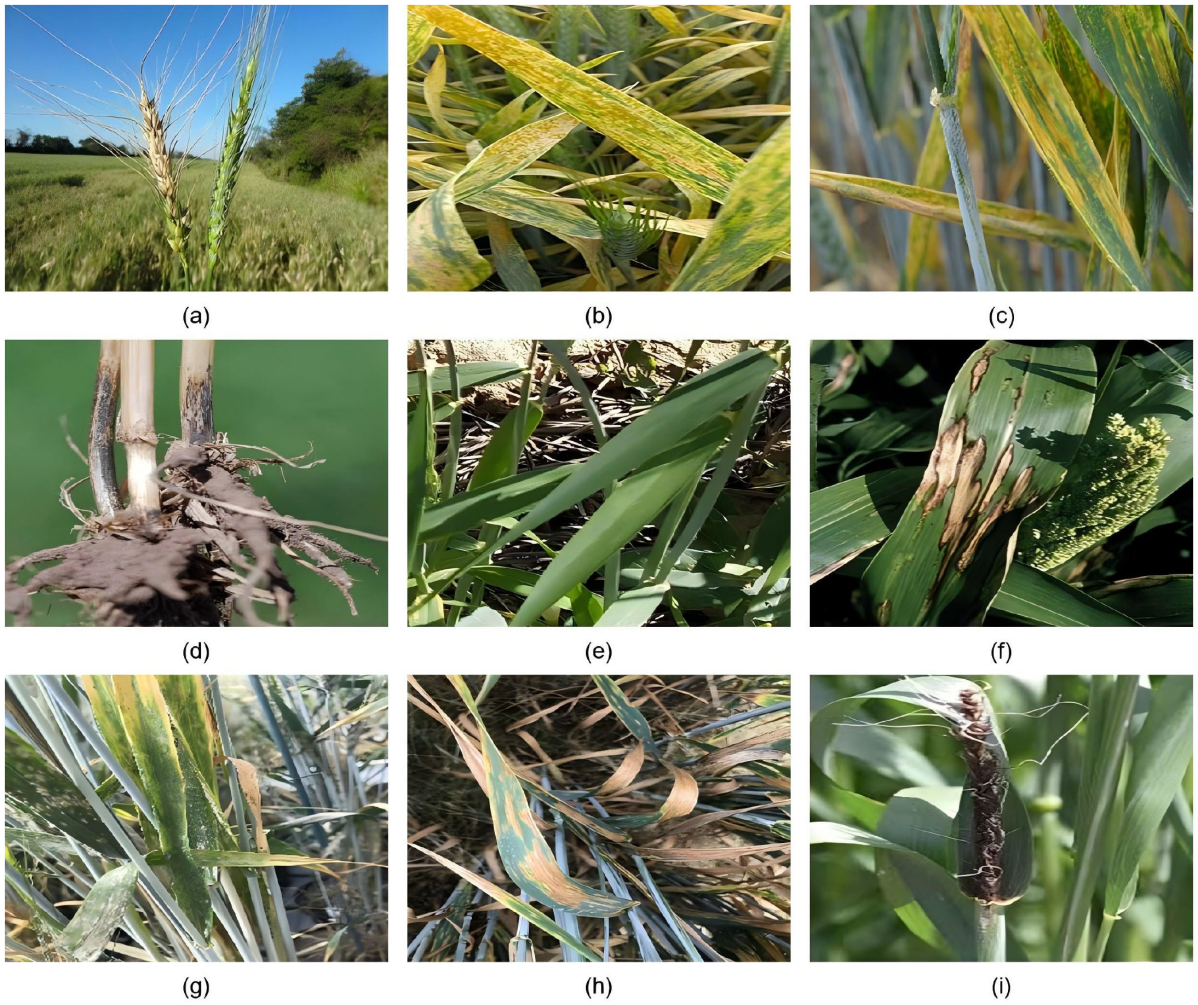
Representative wheat images from the Kaggle dataset: **(a)** Blast, **(b)** Brown Rust, **(c)** Yellow Rust, **(d)** Common Root Rot, **(e)** Healthy, **(f)** Leaf Blight, **(g)** Mildew, **(h)** Septoria, **(i)** Smut. Wheat disease samples constructed from a public Kaggle dataset. Source: https://www.kaggle.com/datasets/kushagra3204/wheat-plant-diseases. License: CC0 (Public Domain Dedication). The images were processed and reorganized by the authors.

The dataset used in this study was obtained from the Kaggle platform. Specifically, the dataset entitled *Wheat Plant Diseases*, released by Kushagra Agarwal, was employed for model development and evaluation. The dataset is publicly available at https://www.kaggle.com/datasets/kushagra3204/wheat-plant-diseases and was accessed on September 5, 2025. The original dataset contains images from 15 disease categories. For this study, eight fungal diseases together with healthy wheat samples were selected for analysis. The numbers of images in each category were 647 for blast, 1,271 for brown rust, 614 for common root rot, 1,000 for healthy wheat, 842 for leaf blight, 1,081 for mildew, 1,144 for septoria, 1,310 for smut, and 1,301 for yellow rust. The dataset was randomly divided into a training set comprising 85% of the samples and a test set containing the remaining 15%. Furthermore, a five-fold cross-validation strategy was employed during model training and evaluation to reduce the bias associated with a single data partition, thereby improving the generalization capability of the model and enhancing the reliability of the experimental results. Detailed information on the dataset is summarized in **Table 1**, while representative images of the selected wheat fungal diseases are presented in **Figure 1**.

**Table 1:** Composition of the wheat dataset.

Wheat disease name	Total images	Training and validation set	Test set
Blast	647	549	98
Brown Rust	1271	1079	192
Common Root Rot	614	521	93
Healthy	1000	850	150
Leaf Blight	842	715	127
Mildew	1081	918	163
Septoria	1144	971	173
Smut	1310	1112	198
Yellow Rust	1301	1105	196

As shown in **Table 1**, noticeable class imbalance exists among the wheat disease categories. Training a model directly on such a dataset may bias the learning process toward majority classes, resulting in degraded classification performance for underrepresented categories. To address this issue, a series of data augmentation strategies, including horizontal flipping, rotation, color jittering, and Gaussian blurring, were applied to enhance sample diversity. Furthermore, a weighted loss function was incorporated during training to compensate for class imbalance and improve model robustness.

### 2.2 The ShuffleNet architecture

**Figure 2:**
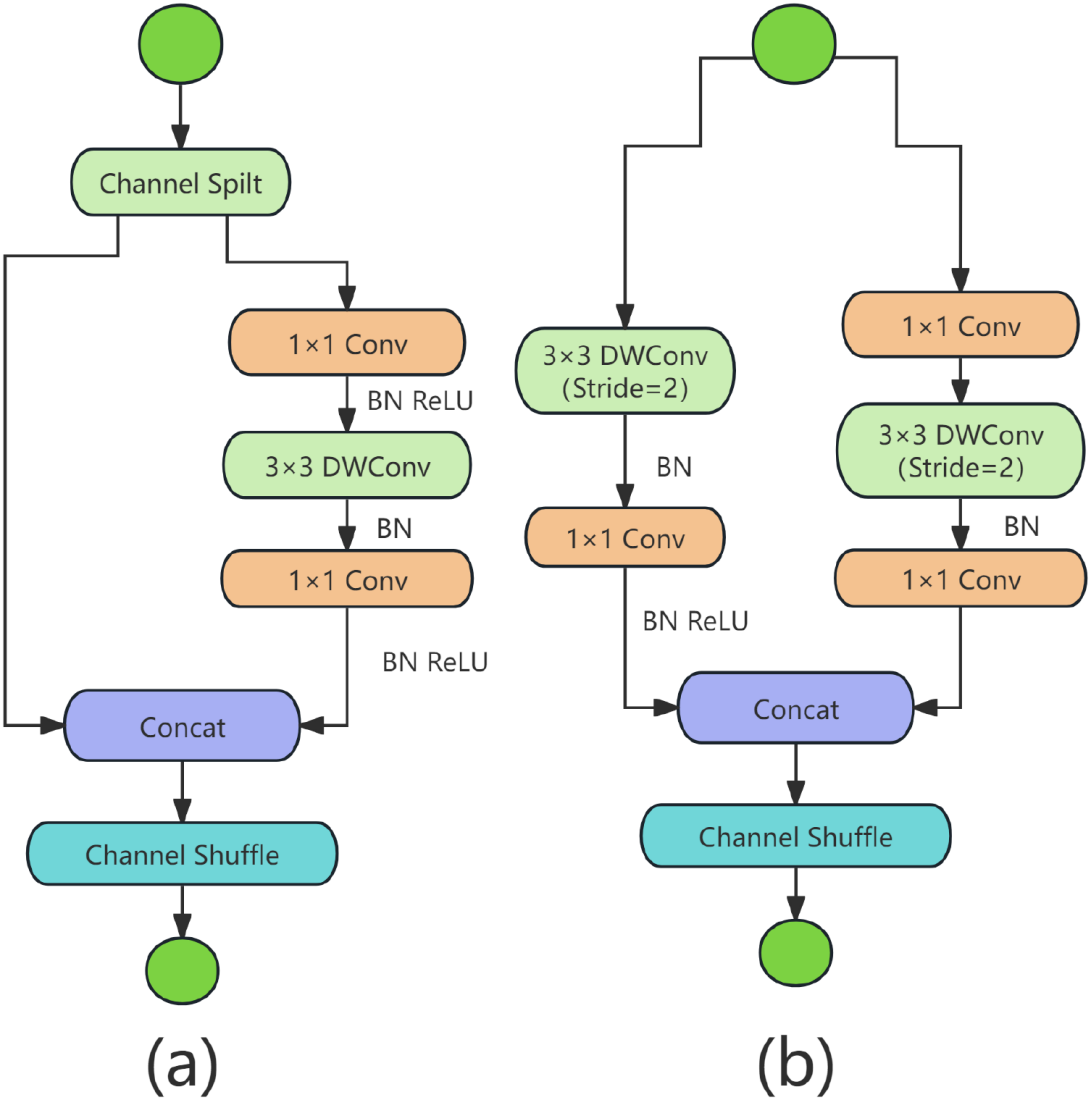
Core module of ShufflenetV2. **(a)** Basic block. **(b)** Dowmsampling block.

In the development of lightweight deep learning architectures, the MobileNet series has become a representative framework owing to its effective reduction of model parameters and computational cost through the introduction of depthwise separable convolutions and inverted residual structures. Although group convolution effectively reduces the number of parameters and computational complexity, it restricts information exchange among channel groups, thereby limiting feature representation capability. To overcome this limitation, Zhang et al. (2018) proposed ShuffleNetV1 in 2017. The network integrates channel shuffle operations with group convolutions, adopts a bottleneck architecture, and replaces standard convolutions with group convolutions to reduce computational cost. By enabling information exchange across different channel groups, the channel shuffle mechanism substantially enhances feature representation while introducing only negligible computational overhead. Nevertheless, ShuffleNetV1 still suffers from several limitations. In particular, pointwise group convolutions incur considerable computational overhead when the channel dimension is small, and certain architectural components leave room for further optimization. To address these issues, ShuffleNetV2 was subsequently proposed. Unlike previous lightweight architectures that primarily focused on reducing floating-point operations (FLOPs), ShuffleNetV2 considers practical factors such as memory access cost, hardware parallelism, and computational efficiency. Consequently, it achieves a better balance between computational efficiency and model accuracy. The core module of ShuffleNetV2 is illustrated in **Figure 2**.

ShuffleNetV2 Ma et al. (2018) introduces a novel building block in which the input feature map is first divided into two branches through a channel split operation. One branch preserves the identity mapping, while the other undergoes a series of convolutional transformations. The outputs of the two branches are subsequently concatenated and processed by a channel shuffle operation to facilitate information exchange across channels. As a representative lightweight architecture, ShuffleNetV2 achieves a favorable balance among theoretical efficiency, inference speed, feature representation capability, and scalability. Unlike the MobileNet series, which primarily focuses on reducing floating-point operations (FLOPs), ShuffleNetV2 takes practical factors such as memory access cost and hardware efficiency into consideration during network design. Consequently, it provides a more effective trade-off between computational cost and recognition performance. Furthermore, its flexible architecture allows the seamless integration of customized modules, making it a suitable backbone for studies involving lightweight network enhancement, attention mechanism incorporation, and multi-scale feature optimization.

### 2.3 Network architecture of ShuffleNetV2 SSM MLCA

**Figure 3:**
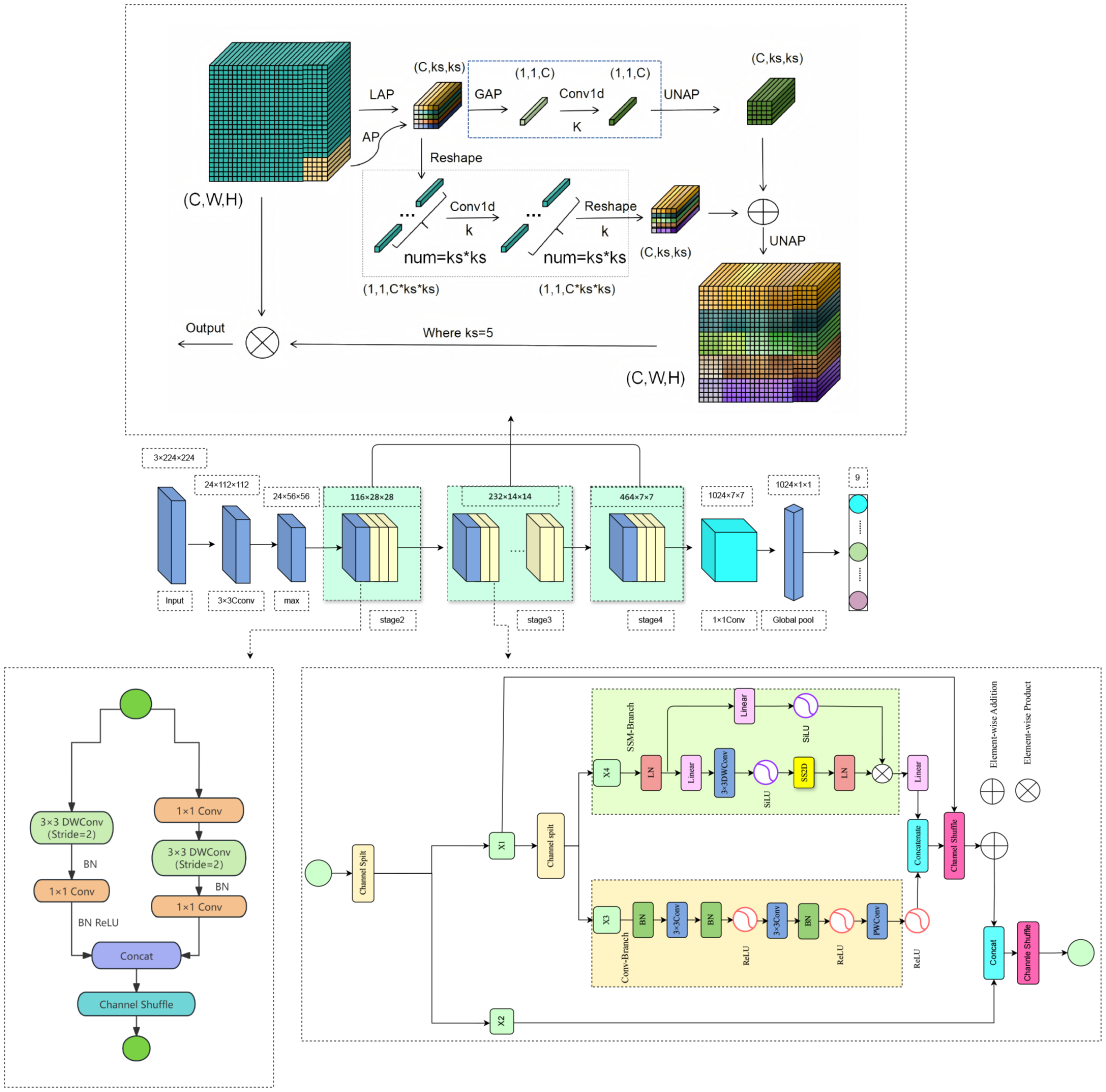
Overall architecture of ShuffleNetV2 SSM MLCA.

As illustrated in **Figure 3**, this study proposes an improved lightweight deep learning model, termed ShuffleNetV2 SSM MLCA, for the recognition of eight common wheat fungal diseases. To overcome the limited receptive field of the original ShuffleNetV2 building blocks, which constrains the extraction of global contextual information, the basic modules are replaced with SS-Conv-SSM modules. This modification enhances the model’s ability to capture both local details and global disease features, thereby improving feature representation capability. Furthermore, to strengthen feature discrimination, a Mixed Local Channel Attention (MLCA) mechanism is embedded into the convolutional branch of the SSConv-SSM module. By adaptively emphasizing informative feature channels and suppressing redundant information, the MLCA module enables the network to focus more effectively on disease-relevant features and further improves recognition performance.

The internal configuration parameters of the ShuffleNetV2 SSM MLCA model are presented in **Table 2**. This table illustrates the variations in feature map size and channel dimension, where n refers to the iteration number of the corresponding operator. Specifically, the stride value is adopted only for the first convolution operation of the operator, while a stride of 1 is utilized for all subsequent operations.

**Table 2:** Detailed network configuration of ShuffleNetV2 SSM MLCA.

Layer index	Operation	Input channel	Output channel	H×W	n	s
0	input	–	3	224×224	1	2
1	conv3×3	3	24	112×112	1	2
2	Bottleneck	24	116	56×56	1	2
3	SS-Conv-SSM	116	116	56×56	3	1
4	Bottleneck	116	232	28×28	1	2
5	SS-Conv-SSM	232	232	28×28	7	1
6	Bottleneck	232	464	14×14	1	2
7	SS-Conv-SSM	464	464	14×14	3	1
8	Bottleneck	464	1024	7×7	1	2
9	GAP	1024	1024	1×1	1	–
10	Linear	1024	*C*	1×1	1	–

### 2.4 SS-Conv-SSM module

**Figure 4:**
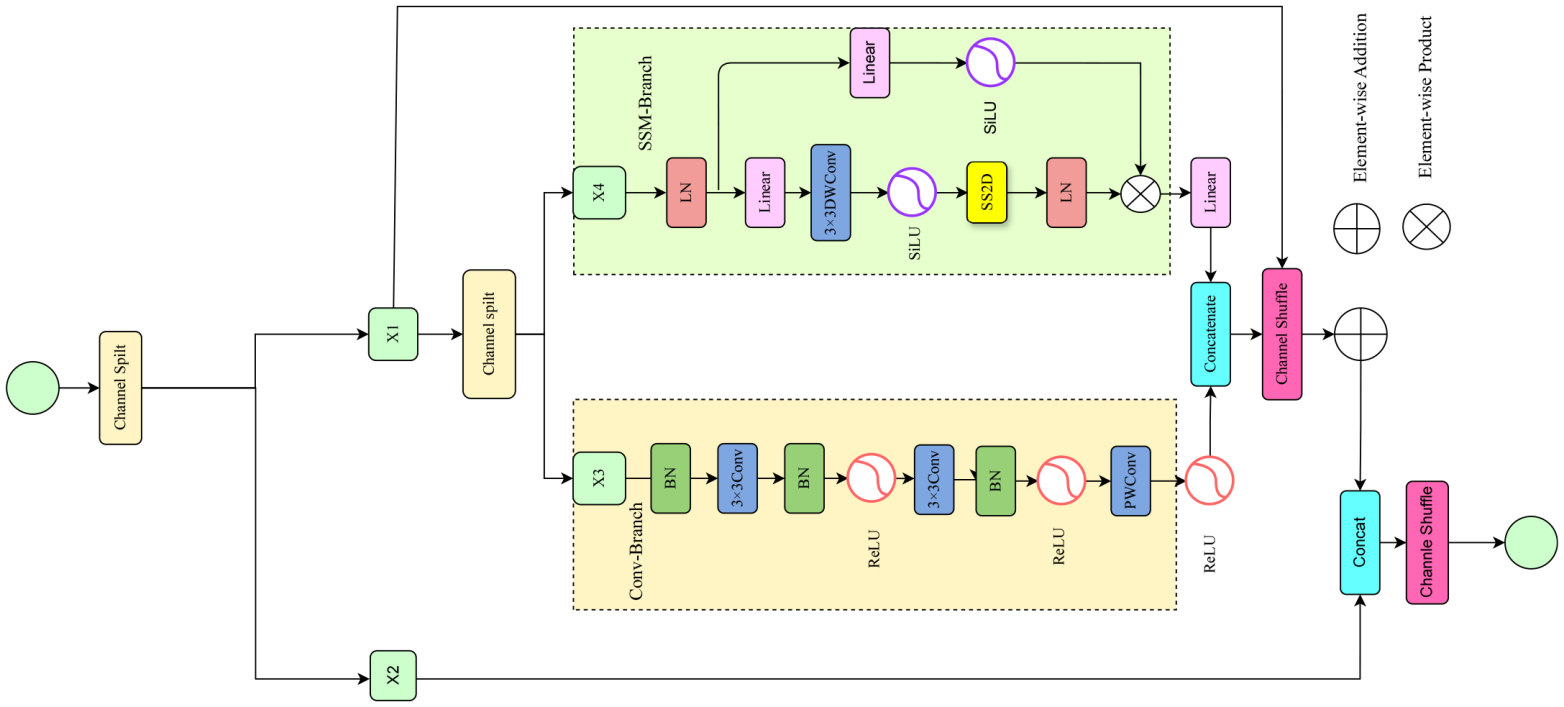
SS-Conv-SSM block.

The SS-Conv-SSM module, proposed in MedMamba Yue and Li (2024), is designed to jointly model local spatial patterns and long-range dependencies in images. By integrating convolutional neural networks (CNNs) with state space models (SSMs), the module effectively combines the strengths of local feature extraction and global context modeling. To further improve computational efficiency, group convolution and channel shuffle operations are incorporated for feature interaction and information exchange. Specifically, the module adopts a dual-branch parallel architecture, where partitioned feature groups are processed separately by a convolutional branch and an SSM branch. The resulting feature representations are concatenated along the channel dimension and shuffled to enhance cross-channel information flow. Furthermore, a residual connection is employed to facilitate gradient propagation and improve training stability. The overall architecture of the SS-Conv-SSM module is shown in **Figure 4**.

The two branches of the SS-Conv-SSM module are designed to capture complementary information and jointly construct multi-level spatial feature representations. The lower branch, namely the convolutional branch, focuses on modeling local spatial patterns through a sequence of convolutional operations. Specifically, the input features are first normalized using batch normalization and then processed by a 3×3 convolution, followed by a ReLU activation function to introduce nonlinearity. Subsequently, an additional convolutional layer is applied to further refine the extracted local features. Finally, a pointwise convolution is employed to facilitate channel-wise feature interaction and information fusion. The computational process of the convolutional branch can be expressed as follows:

First, the input $X\in {\mathbb{R}}^{C\times H\times W}$ undergoes a channel split, yielding $X_{i=1,2}\in {\mathbb{R}}^{C/2\times H\times W}$. *X*_2_ is passed through an identity mapping, while *X*_1_ is fed into the SS-Conv-SSM module. Inside this module, *X*_1_ is further split along the channel dimension into *X*_3_ and $X_{4}\in {\mathbb{R}}^{C/4\times H\times W}$. The final output is $Y\in {\mathbb{R}}^{C\times H\times W}$ (As shown in **Equations 1**–**11**).

(1) $$\bar{\left(X_{3}^{1}\right)}=\text{BatchNorm}\left(X_{3}\right)$$

(2) $$\bar{\left(X_{3}^{2}\right)}=\text{ReLU}\left(\text{BatchNorm}\left({\text{Conv}}_{3\times 3}\left(\bar{\left(X_{3}^{1}\right)}\right)\right)\right)$$

Subsequently, the same sequence of operations—batch normalization, a 3×3 convolution, batch normalization, and ReLU activation—is applied again.

(3) $$\bar{X_{3}^{3}}=\text{ReLU}\left(\text{BatchNorm}\left({\text{Conv}}_{3\times 3}\left(\bar{X_{3}^{2}}\right)\right)\right)$$

A pointwise convolution and ReLU activation are then applied.

(4) $$\bar{\left(X_{3}^{3}\right)}=\text{ReLU}\left(\text{PWConv}\left(\bar{\left(X_{3}^{2}\right)}\right)\right)$$

Meanwhile, the upper branch, referred to as the SSM Branch (SSM-Branch), is responsible for modeling global spatial dependencies. This branch first processes the input features through layer normalization and depthwise separable convolution. Subsequently, a 2D selective scanning (SS2D) module performs a global scan of the feature map along four directions, thereby capturing long-range spatial correlations across pixels. The corresponding mathematical formulation is as follows: First, the input $X_{4}\in {\mathbb{R}}^{\frac{C}{4}\times H\times W}$ is processed by layer normalization:

(5) $$\bar{X_{4}^{1}}=\text{LayerNorm}\left(X_{4}\right)$$

The subsequent steps involve a linear projection, a depthwise separable convolution, and activation via the SiLU function.

(6) $$\bar{X_{4}^{2}}=\text{SiLU}\left(\text{DWConv}\left(\text{Linear}\left(\bar{X_{4}^{1}}\right)\right)\right)$$

It then undergoes the SS2D module to extract global contextual features, followed by layer normalization.

(7) $$\bar{X_{4}^{3}}=\text{LayerNorm}\left(\text{SS}2\text{D}\left(\bar{X_{4}^{2}}\right)\right)$$

Simultaneously, a linear projection and SiLU activation are applied to the original normalized features.

(8) $$\bar{X_{4}^{4}}=\text{SiLU}\left(\text{Linear}\left(\bar{X_{4}^{1}}\right)\right)$$

The SS2D output and the linearly projected output are then multiplied element-wise, followed by integration through a linear layer.

(9) $$\bar{X_{4}^{5}}=\text{Linear}\left(\bar{X_{4}^{3}}⊙\bar{X_{4}^{4}}\right)$$

Finally, the outputs of the two branches are concatenated along the channel dimension and then shuffled.

(10) $$\bar{\bar{X_{1}}}=\text{Shuffle}\left(X_{1}⊕\text{Concatenate}\left(\bar{X_{3}^{4}},\bar{X_{4}^{5}}\right)\right)$$

The feature maps $\bar{\bar{X_{1}}}$ and *X*_2_ obtained from the SS-Conv-SSM module are concatenated along the channel dimension and then shuffled to produce the final output feature map $Y\in {\mathbb{R}}^{C\times H\times W}$.

(11) $$Y=\text{Shuffle}\left(\text{Concat}\left(\bar{\bar{X_{1}}},X_{2}\right)\right)$$

The SS-Conv-SSM module achieves an integrated fusion of local details and global context, forming a comprehensive and highly discriminative spatial feature representation. This design significantly enhances the feature extraction capability of the model in medical image classification tasks while maintaining computational efficiency.

### 2.5 Half convolution

Traditional convolution performs dense computations over all input channels, leading to considerable memory access overhead and computational burden. Although depthwise separable convolution has been widely employed in lightweight neural networks to reduce computational complexity, it often sacrifices feature representation capability and incurs frequent memory access operations, limiting its ability to achieve an optimal trade-off between efficiency and accuracy. To improve the feature extraction capability of the network, the original ShuffleNetV2 building block is replaced with the SS-Conv-SSM module. By integrating convolutional operations with state space modeling, the modified architecture is able to capture both local spatial information and long-range dependencies more effectively. However, the enhanced feature modeling capability comes at the cost of increased parameters and floating-point operations (FLOPs), which weakens the lightweight advantage of the network. Therefore, an efficient lightweight operator is further introduced to reduce computational overhead while preserving the representational power of the model.

**Figure 5:**
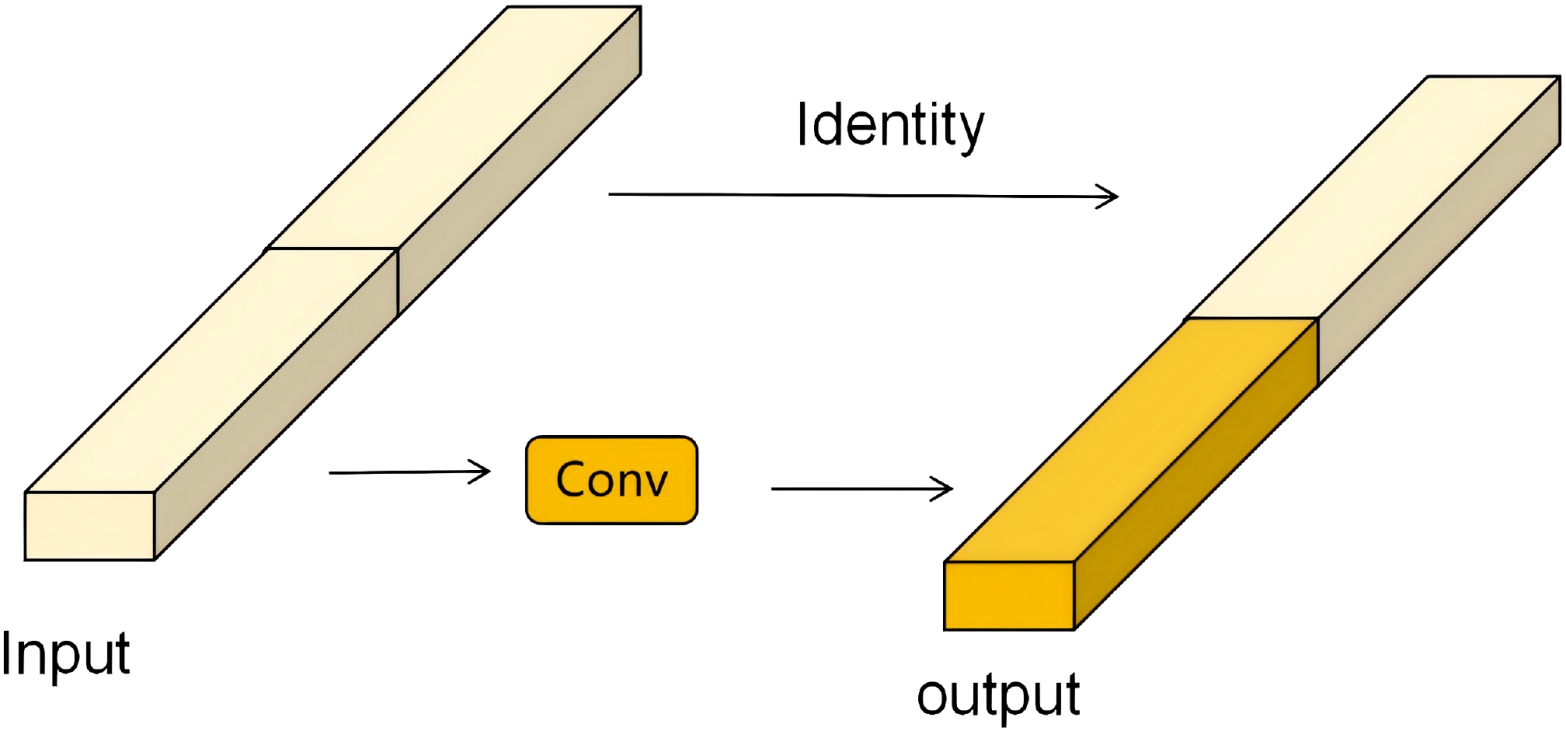
Half convolution.

As illustrated in **Figure 5**, the half convolution operator Liu et al. (2025) adopts a lightweight design in which only half of the input channels participate in convolutional computation, while the remaining channels are directly preserved. The processed and unprocessed feature channels are subsequently concatenated to produce the final output. The corresponding operation can be expressed as follows (**Equation 12**):

(12) $$Y=\text{Concat}\left(\text{Conv}\left(X_{h,w,0,\frac{C}{2}}\right),\;X_{h,w,\frac{C}{2}+1:C}\right)$$

where (X) and (Y) denote the input and output feature maps, respectively, and (C) represents the channel dimension.

By performing convolution on only half of the feature channels, the half convolution operator reduces the FLOPs of conventional convolution by approximately 75% and substantially decreases memory access overhead while maintaining identical input and output dimensions. This design effectively improves computational efficiency and reduces storage requirements without significantly compromising feature representation capability. Moreover, the preserved channels retain original spatial and semantic information, which can be leveraged during subsequent feature fusion to enhance representation diversity. Compared with widely used lightweight strategies, including grouped convolution, channel pruning, and knowledge distillation, half convolution preserves inter-channel information flow more effectively during model compression, thereby mitigating performance degradation. In addition, its deployment does not rely on auxiliary training frameworks or extra optimization procedures, resulting in a straightforward implementation and making it well suited for lightweight network design.

### 2.6 Mixed local channel attention

Mixed Local Channel Attention (MLCA) Wan et al. (2023) is a lightweight attention mechanism designed to improve the spatial perception capability of conventional channel attention modules. Unlike approaches such as CBAM that model channel and spatial attention in a sequential manner, MLCA employs a localglobal dual-path parallel architecture to aggregate information from different receptive fields. Through this design, the network is able to simultaneously capture inter-channel dependencies and local spatial contextual information. In contrast to conventional attention mechanisms that depend on channel dimensionality reduction and global pooling operations, MLCA preserves richer spatial information by partitioning the input feature map into multiple local regions and performing feature refinement within each region. This strategy enhances sensitivity to fine-grained spatial structures while maintaining computational efficiency. Moreover, the module utilizes 1D convolution and unpooling operations to facilitate effective interaction between local features and global semantic information, thereby improving multi-scale feature representation and feature discrimination capability.

**Figure 6:**
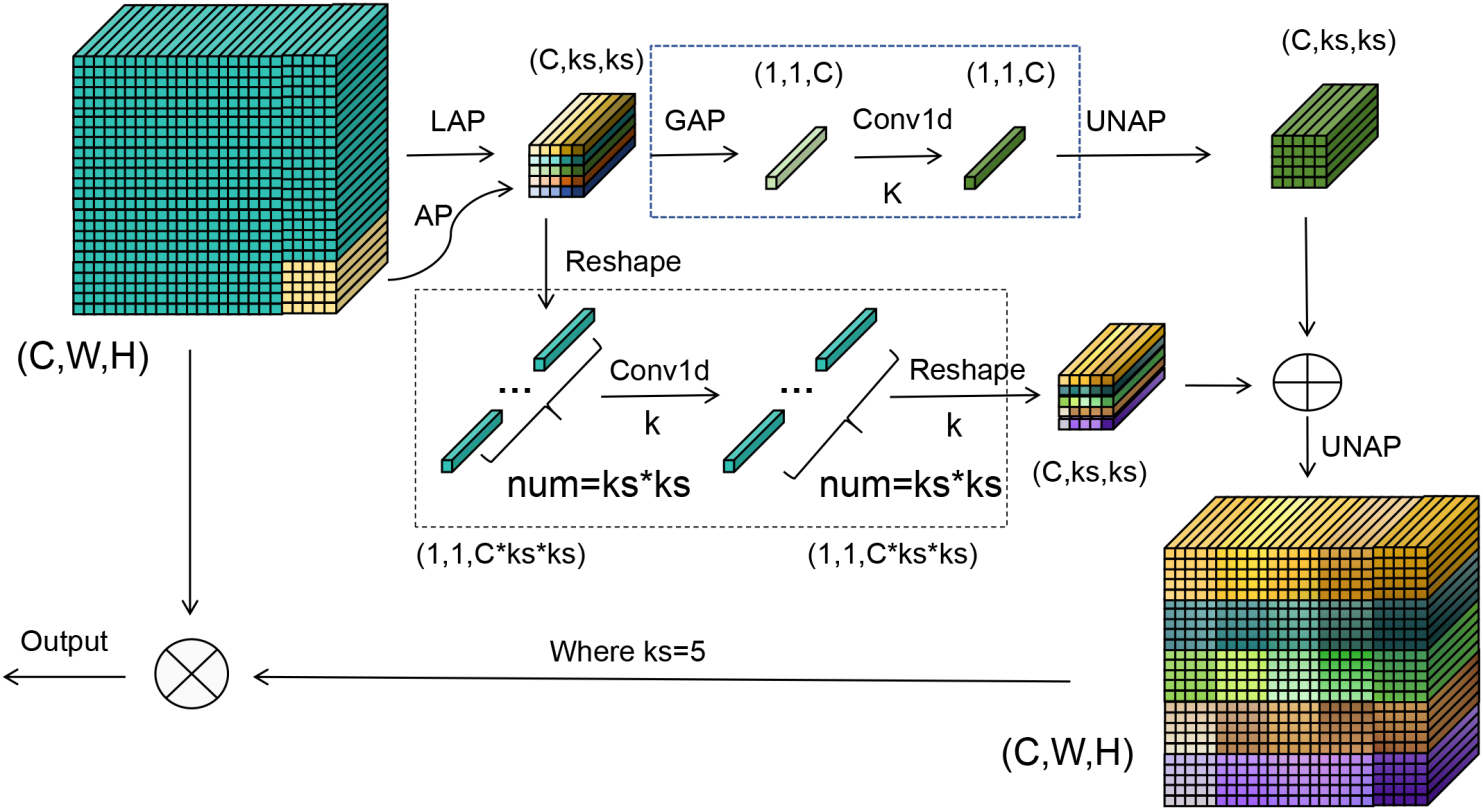
Mixed local channel attention.

As shown in **Figure 6**, MLCA enhances feature representation through three key design mechanisms. First, local average pooling is employed to partition the feature map into multiple spatial sub-regions, enabling region-wise feature refinement and improving the model’s sensitivity to local spatial structures. Second, a dual-branch architecture is adopted to separately capture local spatial information and global channel dependencies, thereby preserving fine-grained spatial details while maintaining global contextual relationships. This design helps alleviate the loss of spatial information commonly associated with attention mechanisms that rely solely on global pooling. Furthermore, 1D convolution is introduced to efficiently model channel interactions, strengthening channel correlations without substantially increasing computational complexity. The resulting features are subsequently restored to the original spatial resolution through an unpooling operation, facilitating effective fusion of local and global information. Collectively, these mechanisms enhance feature representation and discriminative capability while maintaining low computational overhead, demonstrating the effectiveness of MLCA as a lightweight attention module.

## 3 Experiments and results

### 3.1 Experimental setup

To eliminate the influence of experimental conditions on model evaluation and ensure the reliability and reproducibility of the results, all experiments were conducted using the same hardware and software environment. The detailed system specifications are presented in **Table 3**. During training, the random seed was fixed at 42. The initial learning rate was set to 0.001, the batch size was set to 32, the weight decay coefficient was set to 0.0001, and the maximum number of training epochs was fixed at 100. In addition, all input images were resized to 224 × 224 pixels before being fed into the network. To validate the effectiveness of the proposed method, comparative experiments were conducted against several widely used convolutional neural network architectures, including MobileNetV2, ResNet50, Xception, GhostNet, GhostNetV2, EfficientNetB2 M, and MobileNetV3 Large. The performance of all models in wheat fungal disease classification was evaluated using the aforementioned metrics. Furthermore, identical optimization settings and data augmentation strategies were employed across all models to guarantee a fair comparison.

**Table 3:** Training configuration parameters.

Configuration	Parameters
CPU	Intel(R)Core(TM)i5–10500 CPU @3.10GHz
GPU	NVIDIA GeForce RTX 3090
Operating System	Windows 10
Accelerated Environment	CUDA 12.8
Development Environment	Pycharm2023
Deep Learning Framework	PyTorch 2.8.0

### 3.2 Evaluation metrics

A comprehensive evaluation of an image classification model generally encompasses two aspects: classification performance and computational complexity. Classification performance is commonly assessed using metrics such as accuracy, precision, recall, and F1-score, whereas computational complexity is typically quantified in terms of model size, floating-point operations (FLOPs), and parameter count. These metrics collectively reflect the effectiveness of feature learning and the efficiency of model computation.

Model performance is commonly evaluated using metrics such as accuracy, precision, recall, and F1-score. Accuracy measures the proportion of correctly classified samples among all evaluated samples. Precision quantifies the proportion of true positive predictions among all samples predicted as positive, reflecting the reliability of positive predictions. Recall evaluates the model’s ability to identify positive samples, with higher recall indicating a lower rate of missed detections. As precision and recall may exhibit a trade-off relationship, the F1-score, defined as their harmonic mean, provides a balanced assessment of model performance and is particularly suitable for datasets with class imbalance. In addition, the confusion matrix provides a visual representation of the correspondence between predicted and true labels, facilitating the analysis of classification behavior and misclassification patterns. The corresponding definitions of these metrics are given as follows (As shown in **Equations 13**–**16**):

(13) $$\text{Accuracy}=\frac{TP+TN}{TP+TN+FP+FN}$$

(14) $$\text{Precision}=\frac{TP}{TP+FP}$$

(15) $$\text{Recall}=\frac{TP}{TP+FN}$$

(16) $$\text{F}1-\text{score}=2\times \frac{\text{Precision}\times \text{Recall}}{\text{Precision}+\text{Recall}}$$

Where, True Positive (TP) refers to cases where both the predicted result and the true label are positive samples; True Negative (TN) refers to cases where both are negative samples; False Positive (FP) indicates that the predicted result is positive while the true label is negative; and False Negative (FN) indicates that the predicted result is negative while the true label is positive.

Model complexity is typically evaluated in terms of parameter count, computational cost, and model size. The parameter count reflects the representational capacity of the model and is closely associated with its risk of overfitting. Computational complexity is commonly quantified by floating-point operations (FLOPs), which directly affect inference efficiency and computational resource consumption. Model size is determined by both the number of parameters and the numerical precision used for parameter storage, serving as an important indicator of the storage requirements and overall complexity of the model.

### 3.3 Comparison with other models

To comprehensively evaluate the effectiveness of the proposed ShuffleNetV2 SSM MLCA model, comparative experiments were conducted using several representative convolutional neural network architectures, including MobileNetV2, ResNet50, Xception, GhostNet, GhostNetV2, EfficientNetB2 M, and MobileNetV3 Large. All models were trained and evaluated under identical dataset and experimental settings. The evaluation was performed from three perspectives: classification performance, model size, and computational complexity. Classification performance was assessed using four metrics, namely accuracy, precision, recall, and F1-score, while model complexity was evaluated in terms of model size (MB) and floating-point operations (FLOPs).

As shown in **Table 4**, the proposed ShuffleNetV2 SSM MLCA model achieved the highest performance among all compared methods across multiple evaluation metrics. Specifically, it attained an accuracy of 91.35%, outperforming all competing models. Among the lightweight networks, MobileNetV3-Large and GhostNet also achieved relatively high accuracies of 90.00% and 89.35%, respectively. In comparison, larger network architectures such as ResNet50 and EfficientNetB2 M obtained accuracies of 90.65% and 89.42%, respectively, but required substantially greater model sizes and computational costs. Notably, GhostNetV2 achieved a slightly lower accuracy than GhostNet, indicating that architectural modifications do not necessarily lead to performance gains.

**Table 4:** Performance comparison of different models.

Algorithms	Acc(%)	Prec(%)	Rec(%)	F1(%)	Size(MB)	FLOPs(G)	Param(M)
MobileNetV2	82.01	80.61	81.13	80.56	8.73	2.33	2.25
Xception	90.22	88.88	88.6	88.67	80.39	4.67	21
EfficientV2_M	89.42	88.05	87.88	87.95	160.5	2.78	42
GhostNet	89.35	88.29	87.37	87.62	10.52	0.16	2.73
GhostNetV2	88.49	87.26	86.94	87.07	17.59	0.16	4.57
ResNet50	90.65	89.49	89.26	89.32	89.95	4.13	24
MobileNetV3_Large	90	88.74	88.77	88.74	16.14	0.24	4.21
ShuffleNetV2_SSM_MLCA	**91.35**	**90.03**	**89.87**	**89.88**	**10.75**	**0.3**	**2.81**

Bold values denote the results of our proposed model, ShuffleNetV2_SSM_MLCA. For Accuracy, Precision, Recall, and F1‑score, the bolded numbers are the highest among all compared models. For model size (MB), FLOPs (G), and parameter count (M), the bolded values indicate that our model outperforms the majority of the benchmark models, though not necessarily all of them.

In terms of model complexity, ShuffleNetV2_SSM_MLCA achieves a model size of 10.75 MB and requires only 0.3 G FLOPs while maintaining superior classification performance. These results indicate an effective balance between recognition accuracy and computational efficiency. In comparison, although EfficientNetB2_M and ResNet50 achieve relatively high classification accuracies, their model sizes reach 160.5 MB and 89.95 MB, respectively, accompanied by substantially higher computational costs.

In summary, ShuffleNetV2 SSM MLCA achieves a favorable balance between classification performance and computational complexity. The proposed model attains superior wheat fungal disease classification performance while maintaining a lightweight architecture, indicating its effectiveness for accurate and efficient disease recognition.

**Figure 7:**
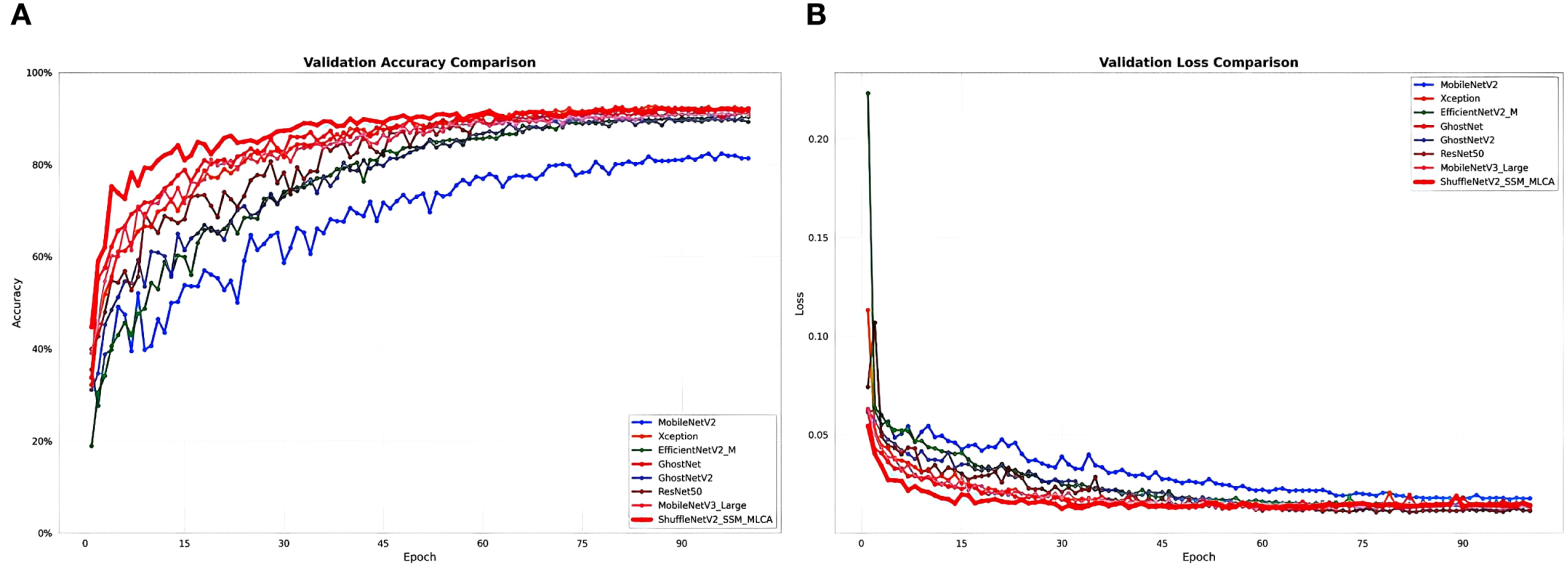
Validation accuracy and loss trends of different models. **(a)** Accuracy. **(b)** Loss.

As shown in **Figure 7a**, the validation accuracy of all models gradually increases with training progress. Among them, the proposed ShuffleNetV2 SSM MLCA model exhibits a faster convergence rate during the early stages of training and progressively outperforms the other architectures as training proceeds. Ultimately, it achieves the highest validation accuracy, suggesting superior feature representation and generalization ability. In contrast, conventional architectures such as ResNet50 and MobileNetV2 demonstrate slower accuracy improvement and less favorable convergence characteristics throughout the training process.

**Figure 7b** presents the validation loss curves of the compared models during training. The proposed ShuffleNetV2 SSM MLCA model exhibits favorable convergence behavior, reaching a stable state earlier than most competing architectures. Although its final validation loss is slightly higher than that of certain models, it achieves superior results across multiple evaluation metrics, including accuracy, precision, recall, and F1-score. This observation may be attributed to the architectural enhancements introduced in the proposed network, particularly the incorporation of multi-scale feature fusion and the Mixed Local Channel Attention (MLCA) mechanism. These components facilitate more effective feature representation and discrimination, thereby improving overall classification performance while maintaining stable optimization characteristics throughout the training process.

### 3.4 Ablation experiment and stability analysis

To evaluate the effectiveness of the proposed components, ablation studies were conducted in this work. The entire dataset was randomly divided into a training set (85%) and a test set (15%) using a fixed random seed of 42, and 5-fold cross-validation was employed to assess the performance improvements and robustness contributed by the proposed SS-Conv-SSM module and Mixed Local Channel Attention (MLCA) mechanism. All experimental settings, including dataset partitioning, hyperparameter configuration, and operating environment, were kept consistent across all comparative experiments. Four widely used evaluation metrics, namely Accuracy, Precision, Recall, and F1-score, were adopted to quantitatively analyze the contribution of each component within the proposed network. The detailed results are presented in **Table 5**.

**Table 5:** Results of ablation tests.

Model	SS-Conv-SSM	MLCA	Accuracy (%)	Precision (%)	Recall (%)	F1-score (%)
shuffleNetv2	×	×	90.19 ± 0.15	88.77	88.87	88.78
shuffleNetv2 SSM	✓	×	91.08 ± 0.25	89.88	89.70	89.74
shuffleNetv2 SSM MLCA	✓	✓	91.35 ± 0.20	90.03	89.87	89.88

As reported in **Table 5**, the proposed modifications consistently improve the performance of the baseline ShuffleNetV2 model. The baseline network achieves an accuracy of 90.19%, a precision of 88.77%, a recall of 88.87%, and an F1-score of 88.78%. When the SS-Conv-SSM module is introduced, the accuracy increases to 91.08%, representing an improvement of 0.89 percentage points, while precision, recall, and F1-score also exhibit corresponding gains. This performance enhancement suggests that the combination of convolutional operations and state-space modeling effectively strengthens the network’s ability to extract both local and global features, thereby improving feature representation quality and classification performance.

Further incorporation of the MLCA module on top of the SS-Conv-SSM module leads to additional improvements across all evaluation metrics. As shown in **Table 5**, the final ShuffleNetV2 SSM MLCA network achieves an accuracy of 91.35% with a standard deviation of 0.20%, while the precision, recall, and F1-score reach 90.03%, 89.87%, and 89.88%, respectively. The observed performance gains, together with the low standard deviation, indicate that the combination of SS-Conv-SSM and MLCA not only enhances classification accuracy but also contributes to stable model performance. By effectively integrating local feature information and global contextual dependencies, the MLCA mechanism enables the network to focus on disease-related discriminative regions while suppressing irrelevant background interference, thereby improving classification robustness under complex field conditions.

The ablation results further demonstrate the complementary roles of the SS-Conv-SSM and MLCA modules. Specifically, the SS-Conv-SSM module enhances feature extraction by jointly modeling local spatial information and long-range dependencies, whereas the MLCA module adaptively emphasizes discriminative disease-related features while suppressing irrelevant background interference. The combination of these two modules effectively improves feature representation quality without compromising the lightweight nature of the original ShuffleNetV2 architecture. Compared with the baseline model, the proposed approach achieves gains of 1.16, 1.26, 1.00, and 1.10 percentage points in accuracy, precision, recall, and F1-score, respectively. The consistent improvements across all evaluation metrics demonstrate the effectiveness of the proposed design and indicate its potential for robust agricultural disease recognition under complex field conditions.

### 3.5 Classification performance for wheat fungal diseases

**Figure 8:**
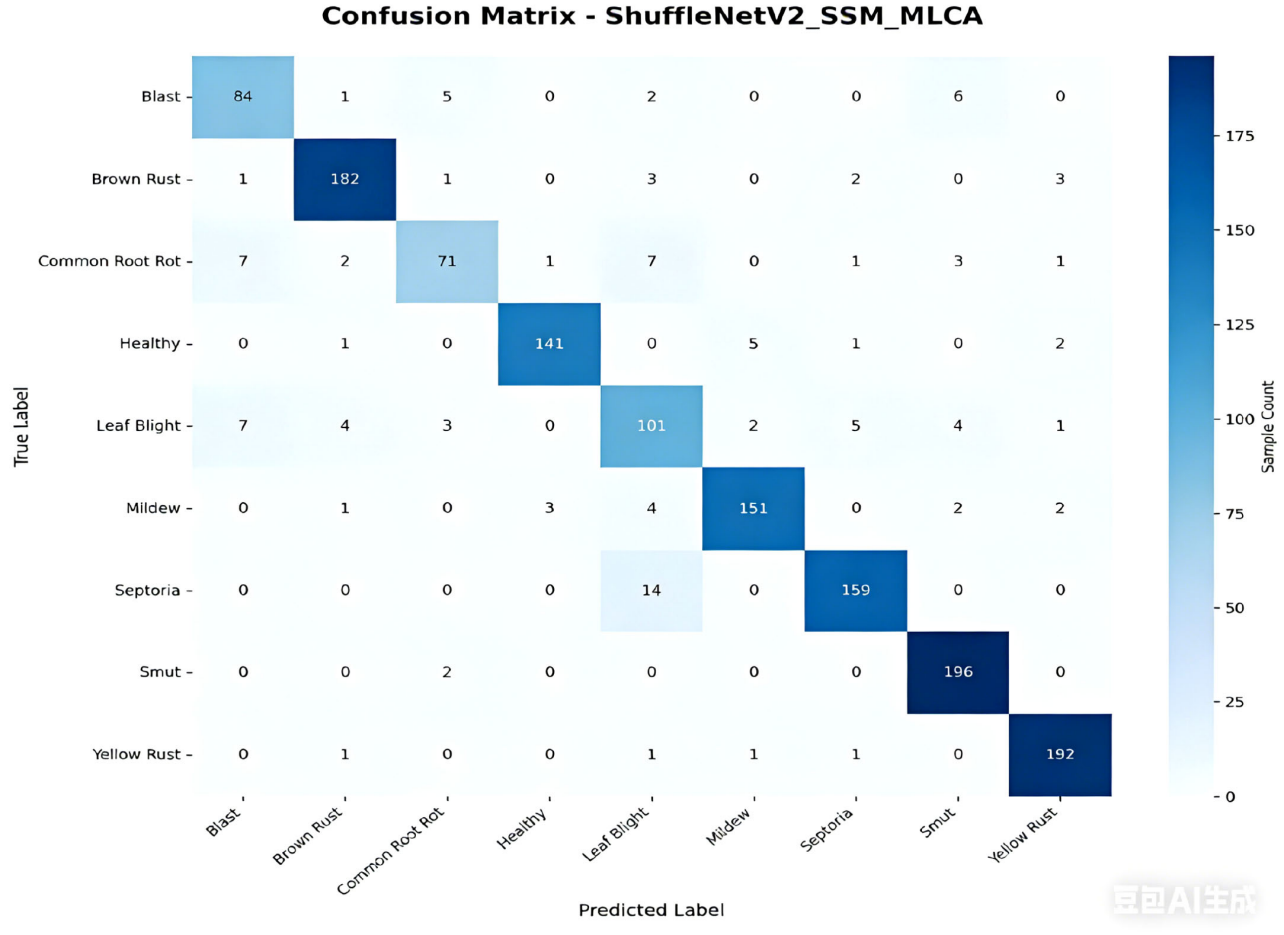
Classification performance of wheat diseases.

To gain deeper insights into the classification behavior of the proposed model, a confusion matrix was generated and is presented in **Figure 8**. The matrix summarizes the prediction results for nine wheat conditions, including eight fungal diseases and healthy plants. Overall, the model achieves high classification accuracy across most categories, particularly for Smut, Brown Rust, Mildew, Septoria, and Healthy samples. The dominance of the diagonal elements indicates that the model is capable of effectively capturing discriminative features and accurately distinguishing among these classes.

Despite the overall satisfactory classification performance, several disease categories still exhibit noticeable confusion. Specifically, among Blast samples, 6 are misclassified as Smut and 5 as Common Root Rot, suggesting that the visual characteristics of these diseases overlap to a certain extent. Similarly, Common Root Rot is frequently confused with Blast (7 instances) and Leaf Blight (7 instances). These misclassification patterns indicate that the affected disease categories share comparable morphological and textural characteristics, which increases the difficulty of accurate classification. Notably, no Leaf Blight samples are incorrectly classified as Healthy, demonstrating that the model can effectively distinguish diseased plants from healthy ones. Furthermore, 5 Healthy samples are misclassified as Mildew, possibly due to background interference or variations in illumination conditions during image acquisition. Overall, these errors can be attributed to the high visual similarity among certain disease symptoms and the influence of complex field environments, both of which pose challenges for discriminative feature extraction and fine-grained classification.

In summary, the proposed model demonstrates strong classification performance across most disease categories, although certain visually similar diseases remain challenging to distinguish. Future work will focus on further enhancing discriminative feature learning to alleviate inter-class confusion. Specifically, a dynamic multi-scale architecture integrated with a soft-gating mechanism could be explored to enable adaptive selection of receptive fields according to lesion scale, thereby improving the representation of disease patterns with varying spatial characteristics. Furthermore, cross-scale feature fusion guided by global contextual information may be introduced to adaptively recalibrate branch-wise attention weights, enhancing the network’s ability to capture both local details and global semantic information. Such improvements are expected to further strengthen the robustness and classification performance of the proposed model.

### 3.6 Analysis of generalization performance

To quantitatively assess the generalization capability of the optimized model, two publicly released wheat disease object detection datasets are employed for experimental validation in this study. The first dataset was published on December 24, 2025 by Baixingdaima on the Alibaba Cloud Developer Community (https://developer.aliyun.com/article/1696461). Built upon field imagery acquired from unmanned aerial vehicle inspections and ground sampling campaigns, it comprises 2000 high-resolution in-field photographs pertaining to four classes, namely barley yellow dwarf, leaf rust, powdery mildew and healthy wheat foliage. The collected samples cover multiple geomorphic types including plains, mountain terraces and salinealkali farmlands under varied seasonal climatic conditions, which faithfully reflect practical field cultivation environments. The second dataset was contributed by blogger DL data set and released on December 25, 2025 via the CSDN Modelers platform (https://modelers.csdn.net/69a67f4c7bbde9200b9c3240.html), containing 10825 field images that document nine wheat diseases such as brown rust and loose smut alongside healthy plant specimens.

Due to overlaps in disease categories and image content between the two datasets, duplicate and highly similar samples were removed before dataset integration. The resulting dataset contains 7,716 images spanning seven categories, and the detailed class distribution is presented in **Table 6**. To ensure a fair comparison, all data preprocessing procedures, training settings, and experimental configurations were kept consistent with those used in the previous experiments.

**Table 6:** Merged wheat disease dataset sample counts.

Wheat disease name	Total images
BarleyYellowDwarf	763
BrownRust	1035
Healthy	1092
LeafRust	1444
PowderyMildew	1526
Septoria	769
YellowRust	1087

As shown in **Table 7**, the proposed ShuffleNetV2 SSM MLCA model achieves an accuracy of 98.56%, a precision of 98.51%, a recall of 98.87%, and an F1-score of 98.67% on the test set. Compared with several representative lightweight convolutional neural networks, including the MobileNet series, Xception, GhostNet, and EfficientNet-M, the proposed model consistently outperforms these architectures across all four evaluation metrics.

**Table 7:** Generalization performance of different models.

Model	Accuracy (%)	Precision (%)	Recall (%)	F1-score (%)
mobilenetv3 large	96.63	96.69	97.30	96.89
xception	96.43	96.34	96.93	96.61
mobilenetv2	96.49	96.57	97.24	96.77
ghostnet	97.53	97.26	97.98	97.59
efficientnetv2 m	97.46	97.23	97.98	97.55
ghostnetv2	94.91	94.96	95.69	95.24
ShuffleNetV2 SSM MLCA	**98.56**	**98.51**	**98.87**	**98.67**

Bold values denote the results of our proposed model, ShuffleNetV2_SSM_MLCA. For Accuracy, Precision, Recall, and F1‑score, the bolded numbers are the highest among all compared models. For model size (MB), FLOPs (G), and parameter count (M), the bolded values indicate that our model outperforms the majority of the benchmark models, though not necessarily all of them.

## 4 Discussion

This study develops a lightweight wheat disease recognition framework by integrating state-space modeling and attention mechanisms into the ShuffleNetV2 backbone. Rather than introducing a fundamentally new network architecture, the proposed ShuffleNetV2_SSM_MLCA represents an effective integration and adaptation of existing lightweight design principles, state-space modeling, and attention-based feature enhancement strategies for wheat fungal disease recognition under complex field conditions.Experimental results demonstrate that such integration significantly improves feature representation capability while preserving the lightweight characteristics of the original backbone network.

The SS-Conv-SSM module combines convolutional operations and state-space modeling through a dual-branch architecture. The convolution branch focuses on extracting local texture and edge information, whereas the SSM branch captures long-range spatial dependencies and global contextual information through selective scanning operations. This complementary design enables the network to simultaneously exploit local discriminative cues and global structural patterns. By overcoming the limited receptivefield of conventional convolutions, the module establishes richer contextual relationships and enhances the discriminative capability of disease features. A similar design philosophy has been reported in recent crop disease recognition studies, which emphasize that the effective integration of local feature extraction and global contextual modeling can substantially improve feature representation and classification performance under complex agricultural environments Paul et al. (2025a).

The Mixed Local Channel Attention (MLCA) module further enhances feature representation by jointly modeling local and global channel dependencies. Through adaptive fusion of fine-grained local attention and coarse-grained global attention, the module improves the network’s ability to focus on disease-relevant regions while suppressing redundant background information. This characteristic is particularly beneficial for wheat disease images, where lesion size, shape, and texture often vary considerably across disease categories. Furthermore, the lightweight implementation based on one-dimensional convolution introduces only a small computational overhead while maintaining strong feature enhancement capability. Similar findings have been reported inrecent lightweight plant disease recognition research, where multi-level attention fusion was demonstrated to effectively strengthen hierarchical feature learning and improve classification robustness under complex field environments.

The overall architecture adopts a progressive feature refinement strategy. Specifically, MLCA modules are selectively incorporated at different network stages according to feature abstraction levels. Shallow layers primarily preserve low-level structural information and therefore employ fewer attention operations to avoid introducing unnecessary feature perturbations. In contrast, deeper layers focus on high-level semantic representations and benefit from enhanced attention-guided feature selection. This staged deployment strategy facilitates efficient utilization of computational resources while maintaining strong discriminative capability. Similar observations have been reported in recent lightweight plant disease recognition studies, where attention mechanisms deployed across multiple feature stages were shown to improve hierarchical feature learning and classification robustness under complex field conditions Paul et al. (2025b). Furthermore, residual connections and channel shuffle operations contribute to stable gradient propagation and effective feature interaction throughout the network.

Despite the favorable classification performance achieved by the proposed framework, several limitations remain. First, the introduction of SS-Conv-SSM and MLCA inevitably increases model complexity relative to the original ShuffleNetV2 backbone.Although half convolution effectively alleviates computational overhead, the sequential computation characteristics of state-space models may still introduce additional memory consumption and inference latency. Second, the model relies on sufficient annotated samples for robust parameter optimization and may exhibit reduced performance when confronted with limited training data or subtle early-stage disease symptoms. Moreover, certain disease categories with highly similar visual characteristics remain challenging to distinguish, as evidenced by the confusion matrix analysis.

Overall, the experimental results demonstrate that ShuffleNetV2_SSM_MLCA achieves superior classification performance while maintaining a lightweight architecture. The model attains an accuracy of 91.87% with a model size of only 10.75 MB. Consistent improvements observed in comparative experiments, ablation studies, five-fold cross-validation, and cross-dataset validation confirm the effectiveness of integrating state-space modeling and mixed local channel attention for wheat disease recognition. These findings indicate that the proposed framework provides an effective and computationally efficient solution for disease identification in complex agricultural environments.

## 5 Conclusions

To address wheat fungal disease recognition in complex field environments, this study proposes a lightweight classification network, ShuffleNetV2 SSM MLCA, by integrating the SS-Conv-SSM module and the Mixed Local Channel Attention (MLCA) mechanism into the ShuffleNetV2 architecture. The SS-Conv-SSM module enhances feature extraction by jointly modeling local spatial information and long-range dependencies, while the MLCA mechanism further improves feature discrimination through adaptive attention allocation. Experimental results show that the proposed model achieves an accuracy of 91.87% while maintaining low computational complexity and a lightweight architecture. In addition, the model demonstrates favorable generalization capability on cross-dataset validation tasks. Although certain disease categories with highly similar visual symptoms remain difficult to distinguish, the overall results confirm the effectiveness of the proposed design for wheat fungal disease recognition. Future research will investigate more advanced multi-scale feature learning strategies and joint disease localization–classification frameworks to further improve recognition performance in complex agricultural environments.

## Data Availability

The original contributions presented in the study are included in the article/supplementary material. Further inquiries can be directed to the corresponding author.
